# Invasion promotes invasion: Facilitation of C_3_ perennial grass dominance in mixed C_3_/C_4_ grassland by an invasive C_3_ woody sprouter (*Prosopis glandulosa*)

**DOI:** 10.1002/ece3.5800

**Published:** 2019-11-05

**Authors:** Robert James Ansley, Caitlyn Cooper, Tian Zhang

**Affiliations:** ^1^ Natural Resource Ecology and Management Department Oklahoma State University Stillwater OK USA; ^2^ Texas A&M AgriLife Research and Extension Center Vernon TX USA

**Keywords:** coppice, drought, grassland restoration, rangeland, resprouting, shrubs, state‐and‐transition, woody plant encroachment

## Abstract

1. In the Southern Great Plains (SGP) of the United States, encroachment of the native invasive woody legume, honey mesquite (*Prosopis glandulosa* Torr.), has caused a decline in C_4_ mid‐grass abundance. *Prosopis glandulosa* invasion has also facilitated growth of the C_3_ mid‐grass species, Texas wintergrass (*Nassella leucotricha* [Trin & Rupr.] Pohl) initially beneath its canopy but extending to interspaces between *P. glandulosa* as stand density increases. Little is known about the stability of the *Prosopis*/*Nassella* association or C_4_ grass recovery following *P. glandulosa* disturbance.
2. We quantified C_3_ and C_4_ grass production in interspaces, and basal cover in interspaces and *P. glandulosa* subcanopy microsites for 9 years following *P. glandulosa* suppression (top‐kill) and compared this to untreated *P. glandulosa* woodland (woodland).
3. The *Prosopis*/*Nassella* association limited the window of C_4_ mid‐grass recovery to only a few years. *Nassella leucotricha* dominated grass production during the first 3 years after top‐kill. C_4_ mid‐grass recovery began in year 4, but was interrupted by severe drought in years 5 through 7. Recovery resumed in year 8, due to above‐average summer rainfall, but *P. glandulosa* regrowth was large enough by this time to limit C_4_ mid‐grass production to a third of its potential.
4. *Nassella leucotricha* basal cover remained dominant and stable in woodland subcanopy microsites, even during drought, and only briefly declined in top‐kill subcanopy microsites before returning to pretreatment levels by year 8 as *P. glandulosa* regrowth increased and provided shade.
5. *Synthesis and applications*. A single suppression event had little impact on disrupting the *Prosopis*/*Nassella* association and allowing C_4_ mid‐grass recovery. The coupling of a deciduous, N‐fixing C_3_ woody species with this C_3_ perennial grass may be a vegetative “state” that is resistant to multiple woody suppression disturbances and permanently limits the transition back to C_4_ grassland.

## Graphical abstract

We quantified C4 grass recovery for 9 years following woody shrub (Prosopis) suppression (top‐kill). Prosopis regrowth, growth of associated C3 grass (Nassella) beneath the Prosopis canopy, and drought limited the window of C4 grass recovery to only a few years. Thus, a single suppression event had little impact on disrupting the Prosopis/Nassella association and allowing restoration of C4 grasses that were dominant prior to Prosopis invasion.

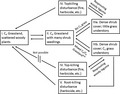

## 1 INTRODUCTION

Many studies have addressed the dynamics of C_3_ and C_4_ plants as functional groups in response to disturbances or predicted changes in atmospheric CO_2_ levels or climate (Bond & Midgley, 2000, 2012; Collatz, Berry, & Clark, 1998). Often these studies have focused on the problem of C_4_ grasslands being displaced by C_3_ woody plants that gain a competitive advantage under increasing CO_2_ levels, lower fire frequency due to anthropogenic activities that suppress natural wildfires, and overgrazing of C_4_ grasses by domestic livestock (Bond, Woodward, & Midgley, 2005; Briggs et al., 2005; Midgley & Bond, 2015). Some of the most invasive woody plants are N‐fixing legumes that eliminate the advantage C_4_ grasses have over C_3_ grasses under frequent fire regimes that lower soil N availability (Prober, Thiele, Lunt, & Koen, 2005; Sage & Kubien, 2003).

Secondary to these processes, and presented less frequently in the literature, is the response of C_3_ grasses when C_3_ woody plants have invaded either mixed C_3_/C_4_ grasslands or C_4_ grasslands where exotic C_3_ grass species have invaded. Typically, C_3_ grasses first become dominant beneath the woody canopies (hereafter “subcanopy”), then increase in spaces between trees (hereafter “interspaces”) as woody plant density increases, all to the detriment of C_4_ grasses. An important example of this process can be found in the Southern Great Plains (SGP), USA, where mixed C_3_/C_4_ grasslands have become invaded by the woody legume, honey mesquite (*Prosopis glandulosa* Torr.), and the graminoid understory has become dominated by the C_3_ mid‐grass, Texas wintergrass (*Nassella leucotricha* [Trin & Rupr.] Pohl; Laxson, Schacht, & Owens, 1997; Simmons, Archer, Teague, & Ansley, 2008). Similar situations of woody species facilitating C_3_ grass growth in C_4_ grasslands have occurred elsewhere, for example, Argentina (Rauber, Steinaker, Demaria, & Arroyo, 2014; Rossi & Villagro, 2003), South Africa (Stuart‐Hill & Tainton, 1989), and Australia (Prober et al., 2005).

*Prosopis glandulosa* is native to the SGP but remained in low densities before European settlement due to frequent fire (ignited mostly by lightning and Native Americans) and competition by grasses (Van Auken, 2000). Loss of fire, overgrazing of grass, and enhanced seed dissemination by cattle via endozoochory are some of the reasons given for *P. glandulosa* expansion (Ansley, Pinchak, & Owens, 2017; Archer, 1989; Brown & Archer, 1989).

Soil isotope research has shown that many areas in the SGP now dominated by woody plants were once mostly C_4_ grasslands (Liao, Boutton, & Jastrow, 2006). Since *N. leucotricha* is identified as being native to the SGP (Stubbendieck, Hatch, & Dunn, 2017; Tyrl, Bidwell, Masters, & Elmore, 2008), we assume it was present in small amounts in mostly C_4_ grasslands prior to *P. glandulosa* invasion and advanced after *P. glandulosa* became dominant. Currently, in a typical dense stand of *P. glandulosa*, *N. leucotricha* is dominant in subcanopy and is mixed with C_4_ grasses in interspaces (Simmons et al., 2008).

*Nassella leucotricha* persists within deciduous *P. glandulosa* stands by maximizing photosynthesis and growth in early spring before *P. glandulosa* leaves emerge and becomes dormant in summer when *P. glandulosa* is in full foliage (Hicks, Briske, Call, & Ansley, 1990; Teague et al., 2014). Dormancy also enables *N. leucotricha* to avoid competition with *P. glandulosa* for soil moisture during summer drought. In contrast, C_4_ grasses must compete directly for light and soil moisture under full‐foliaged *P. glandulosa*. In interspaces where light is available, *P. glandulosa* competes with grasses for soil moisture because it possesses an extensive network of lateral roots that extend into interspaces (Ansley, Boutton, & Jacoby, 2014). C_4_ mid‐grass production rapidly declines once *P. glandulosa* stand‐level canopy cover exceeds 30% (Ansley et al., 2004).

Maintaining dominance of C_4_ mid‐grasses in the SGP is important because they provide the greatest amount of high‐quality forage for cattle (Ansley, Mirik, Heaton, & Wu, 2013) and other ecosystem services such as ground‐nesting bird habitat (Tomecek, Pierce, Reyna, & Peterson, 2017) and carbon sequestration (Liao et al., 2006). Numerous studies have demonstrated that anthropogenic treatment of *P. glandulosa* increases grass production (Bedunah & Sosebee, 1984; Laxson et al., 1997). Because treatments that can completely kill *P. glandulosa* (i.e., “root‐kill”) are expensive (e.g., root‐plowing, $750–900 ha^−1^; aerial spraying root‐killing herbicide, $85–100 ha^−1^), less expensive treatments (e.g., chaining, $30 ha^−1^; prescribed fire, $15 ha^−1^) have often been used that only “suppress” *P. glandulosa* by killing aboveground tissue (i.e., “top‐kill”; Ansley et al., 2004). Top‐killing stimulates multistemmed regrowth that can become very competitive with grasses due to increased stem and leaf density (Ansley, Mirik, & Castellano, 2010).

We know very little about how suppression of *P. glandulosa* affects interactions between C_3_ and C_4_ grass species in mixed grasslands. Since most *P. glandulosa* woodlands with a primarily *N. leucotricha* understory were once C_4_ grassland, the removal or continual suppression of *P. glandulosa* should shift grass production and composition toward C_4_ grasses (Laxson et al., 1997), but few studies have demonstrated this. Because the *P. glandulosa* canopy has such a profound effect on C_3_ and C_4_ grass distribution, the delineation of grass responses within microsite locations following suppression is critical (Ansley et al., 2004).

The *Prosopis*/*Nassella* association, or similar C_3_ shrub/C_3_ grass associations, may alter our view of state‐and‐transition dynamics in semi‐arid savanna ecosystems (Briske, Fuhlendorf, & Smeins, 2003; Westoby, Walker, & Noy‐Meir, 1989). Because *P. glandulosa* is a prolific resprouter, it is uncertain whether resumption of the presettlement mechanism of frequent suppression by fire could shift the *Prosopis*/*Nassella* association back to C_4_ dominant grassland (Ansley, Boutton, Mirik, Castellano, & Kramp, 2010). Moreover, few if any models have considered subcanopy/interspace microsite responses.

Our objective was to quantify responses of the three main perennial grass functional groups in the SGP, C_3_ mid‐grasses, C_4_ mid‐grasses, and C_4_ short‐grasses, to the suppression and subsequent resprouting of *P. glandulosa*. We hypothesized that C_4_ mid‐grasses would respond favorably to *P. glandulosa* suppression but wished to better define the level and extent of recovery before *P. glandulosa* regrowth again becomes dominant. In addition, because the dynamics of C_3_ and C_4_ grasses depend on proximity to *P. glandulosa* canopies, we measured responses of these grass groups within interspace and subcanopy microsite locations.

## 2 MATERIALS AND METHODS

### 2.1 Study site and experimental design

Research was conducted on the Smith‐Walker Experimental Ranch in north central Texas (34°01′52″N; 99°15′00″E; elevation 372 m) which is the north–south mid‐point in the SGP. Mean annual rainfall (30 years; 1981–2010) is 710 mm with peak rainfall in the months of June (108 mm) and September (80 mm). Mean annual air temperature is 17.1°C and monthly air temperatures range from an average daily maximum of 35.9°C in July to an average daily minimum of −2.4°C in January (National Oceanic & Atmospheric Administration‐National Climatic Data Center [NOAA‐NCDC], 2019). Growing season is from mid‐March through October (~240 days). Soils are fine, mixed, superactive, thermic Typic Paleustalfs of the Wichita series that are 1 to 2‐m deep clay loams on 1%–3% slopes with an “Ecological Site designation of Clay loam 23–30” R078CY096TX (United States Department of Agriculture‐Natural Resource Conservation Service [USDA‐NRCS], 2019).

The site was dominated by a *P. glandulosa* overstory (details below). The herbaceous layer was comprised mostly of C_3_ perennial mid‐grass, *N. leucotricha*, and C_4_ perennial short‐grass, buffalograss (*Buchloe dactyloides* [Nutt.] Engelm.). C_4_ perennial mid‐grass species lightly scattered on the site included sideoats grama (*Bouteloua curtipendula* [Michx.] Torr.), vine mesquite (*Panicum obtusum* Kunth), and sand dropseed (*Sporobolus cryptandrus* [Torr.] A. Gray). Common annual grasses were Japanese brome (*Bromus japonicas* Houtt.) and little barley (*Hordeum pusillum* Nutt.). Common forbs were western ragweed (*Ambrosia psilostachya* DC) and annual broomweed (*Amphiachyris dracunculoides* [DC.] Nutt.; Stubbendieck et al., 2017; Tyrl et al., 2008).

*Prosopis glandulosa* was mechanically top‐killed in four 0.5‐ha plots (hereafter “top‐kill” treatment) in October 2006 using a custom‐built machine that cut all vegetation to within 3 cm of the soil surface and removed all but small *P. glandulosa* wood fragments (1–10 cm long; 0.5–2 cm wide) from the site. There was no damage to grasses or soils. *Prosopis glandulosa* height, density, and stand canopy cover before top‐kill were 4.3 m, 578 trees/ha, and 71%, respectively (Ansley, Zhang, & Cooper, 2018). Interspace cover was 29%. The top‐kill treatment reduced *P. glandulosa* stand canopy cover and canopy area of each tree to zero. All *P. glandulosa* survived top‐kill and subsequently resprouted from stem bases. In addition, four 0.5‐ha plots were randomly established in untreated *P. glandulosa* woodland (hereafter “woodland” treatment). Height, density, and cover were 4.0 m, 622 trees/ha, and 66%, respectively. All references to “treatment(s)” in this paper refer to the top‐kill and woodland treatments. Cattle grazed in both treated and untreated plots year‐round during the study period at a stocking rate of 6–8 ha animal unit^−1^ year^−1^ with occasional 3–6 month deferments during droughts.

### 2.2 Data collection

Grass production and basal cover measurements were made in both treatments for each of 9 years after the top‐killing treatment (2007–2015). Because the area was open to grazing by cattle, grass production was measured within five 1 m wide × 2 m long × 1.2 m tall wire cages that were randomly located in interspaces between *P. glandulosa* trees in each plot in February of each year before grass growth began. The interspace area in the top‐kill treatment included the original pretreatment interspace as well as approximately a 1 to 2‐m wideband of what used to be outer subcanopy areas of top‐killed *P. glandulosa*, but did not include the area within a 1‐m border around the cut base stem stumps of any top‐killed tree. Since we could not position similar cages in subcanopy microsites in the top‐kill treatment without potentially disturbing *P. glandulosa* regrowth, comparisons of grass responses between interspaces and subcanopy microsites in each treatment were only assessed using nondestructive basal cover measurements.

To better capture peak production for each functional group, sampling occurred within the cages in early summer (May or June), and in fall (October), after what are typically the greatest growth periods for C_3_ and C_4_ grasses, respectively. All standing herbaceous material within a 0.25‐m^2^ frame was clipped to within 3 cm of ground level and separated into 6 functional groups: C_3_ annual grasses, C_3_ mid‐grasses, C_4_ mid‐grasses, C_4_ short‐grasses, and other forbs. All of the C_3_ mid‐grass samples were *N. leucotricha*. Herbaceous and woody litter were collected from the soil surface within each 0.25‐m^2^ frame. Herbaceous litter included any dead material that had separated from the living plant and had fallen to the ground. Any dead or semisenescent leaf material still connected to the living plant was counted as plant production and not litter. The 0.25‐m^2^ frame was placed in a different location in each cage for the two sampling periods.

Grass and forb samples were oven dried at 60°C, weighed and reported as summer and fall values. We also report production of two variables as combinations of grass functional groups: total perennial grass (TPG = C_3_ mid‐grass + C_4_ mid‐grass + C_4_ short‐grass) and C_4_ perennial grass (C4P = C_4_ mid‐grass + C_4_ short‐grass). Peak values of each functional group from the summer or fall clip date were used to calculate TPG and C4P.

*Prosopis glandulosa* wood fragments were manually removed from each litter sample. Remaining material was filtered with a 2‐mm mesh screen to remove soil. Wood litter (WDLIT) and total herbaceous litter (THLIT) portions were oven dried and weighed. The percentage of *P. glandulosa* leaves by weight in each THLIT sample was visually estimated and subtracted from the total weight to estimate grass + forb litter (GFLIT) production.

Basal cover of each grass species, forbs, litter, and bare ground as a percentage of the total area was visually estimated in the fall each year within a 0.25‐m^2^ wire frame placed at 5‐m intervals along a randomly located line transect in each plot. Grass cover included the area within the circular perimeter of a bunchgrass or irregular patch of rhizomatous grass, not including any gaps that were >25 cm^2^. Cover of perennial grass species was grouped into C_3_ mid‐grasses, C_4_ mid‐grasses, or C_4_ short‐grasses, and, by addition, C4P and TPG. Each of these groups as a percentage of TPG was calculated. Additionally, the percent difference (Pdiff) in mean basal cover of C4P and TPG in top‐kill (TK) versus woodland (WD) in both microsites each year was calculated as:

(1) $$\text{Pdiff}=\left[\left(\text{CoverTK}-\text{CoverWD}\right)/\text{CoverWD}\right]*100$$

Litter cover (hereafter: “total litter cover”) included dead grass and forbs, *P. glandulosa* leaves and wood fragments, and C_3_ annual grasses that had grown in spring and senesced by fall. Precipitation was recorded on site. *Prosopis glandulosa* and soil moisture responses are found in Ansley et al. (2018).

### 2.3 Data analysis

Grass and litter production data (measured only in interspaces) were analyzed per sample date using a completely randomized analysis of variance design with two treatments (top‐kill and woodland) and four replicate values per treatment (SAS, 2013). For basal cover, two analyses were conducted. First, for each functional group, treatments (top‐kill vs. woodland) were compared per sample date within each microsite location (interspace and subcanopy; *n* = 4). Second, microsite effects were compared per sample date within each treatment (*n* = 4). For all response variables, subplot values were averaged to generate replicate plot values. Percentage data were Arcsine transformed prior to analysis. Mean separation was performed using LSD (*p* ≤ .05).

## 3 RESULTS

### 3.1 Precipitation

**Figure 1:**
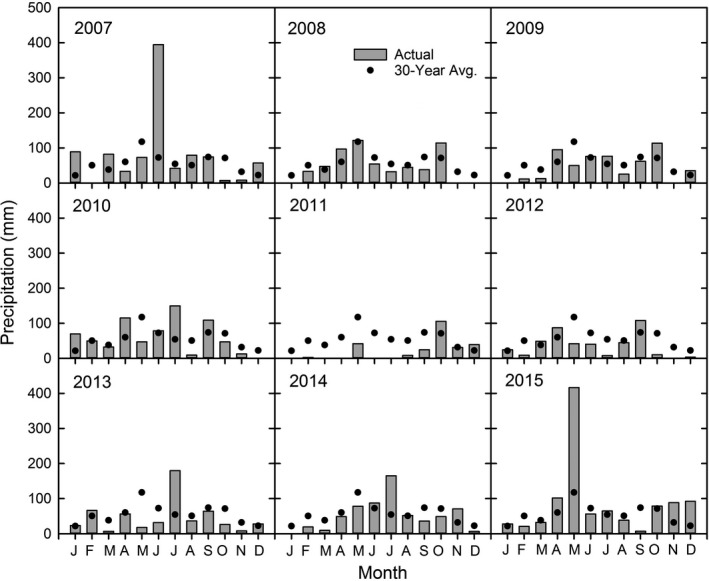
Monthly precipitation totals at the site in 2007–2015, compared to the 30‐year average (Avg.; NOAA‐NCDC, 2019)

Precipitation in the first 4 years after top‐kill was near normal except for 444% and 175% above average precipitation in June 2007 and July 2010, respectively (Figure 1). Extreme drought occurred from 2011 through mid‐2013 until the site received 230% above average precipitation in July 2013, 203% above average precipitation in July 2014, and 254% above average precipitation in May 2015.

### 3.2 Treatment effects on herbaceous production

**Figure 2:**
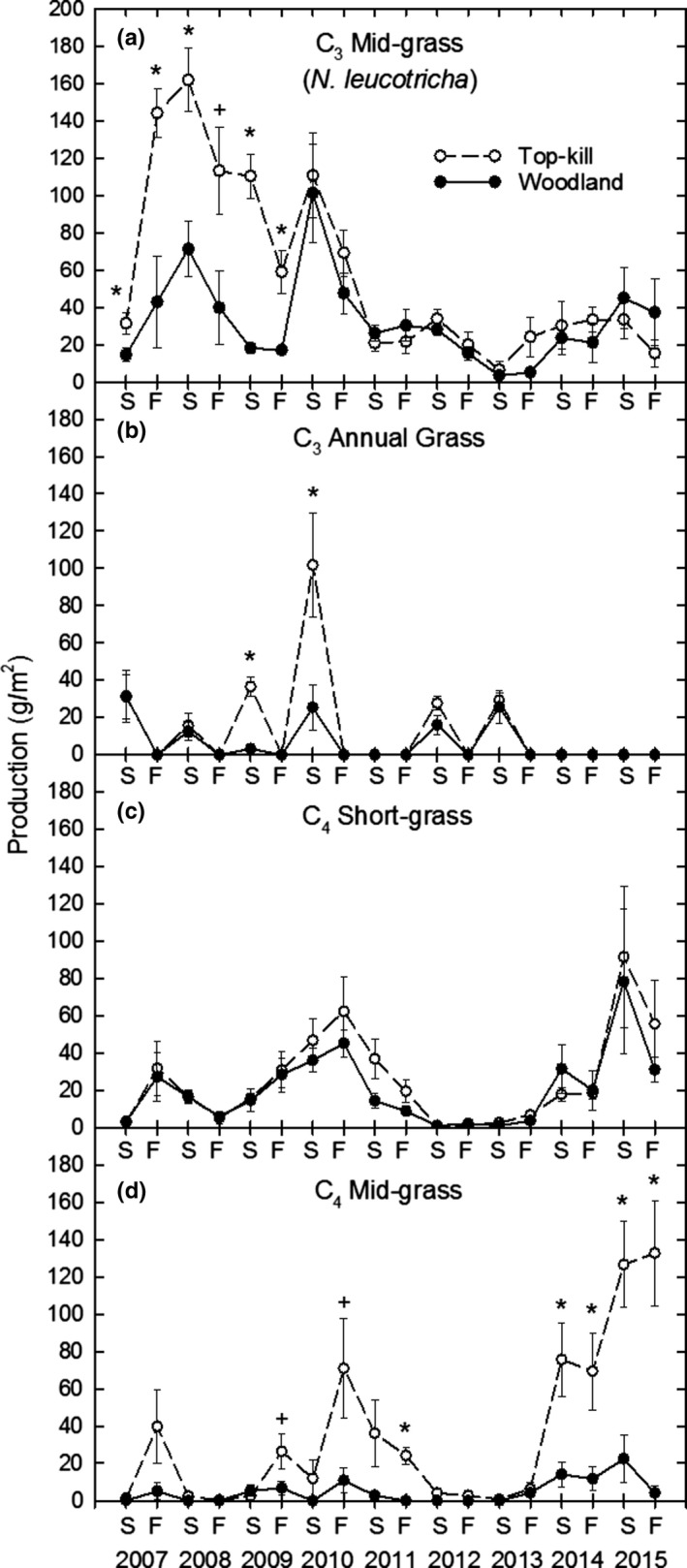
Production of C_3_ mid‐grass (a), C_3_ annual grass (b), C_4_ short‐grass (c) and C_4_ mid‐grass (d) Functional groups in interspaces in top‐kill and woodland treatments by summer (S) and fall (F) each year. Asterisk indicates difference at *p* ≤ .05 for each paired comparison; plus sign indicates difference at *p* ≤ .10

C_3_ mid‐grass production was 2–3 times greater in the top‐kill than the woodland treatment during the first 3 years after *P. glandulosa* top‐kill but was not different between treatments during the rest of the study (Figure 2a). For both treatments, C_3_ mid‐grass production declined to its lowest point in summer 2013, then increased from fall 2013 through summer 2015. C_3_ annual grass production occurred only in spring and was greater in top‐kill than woodland in 2009 and 2010 (Figure 2b). C_4_ short‐grass production remained similar in both treatments throughout the study, with peak production in 2010 and 2015 (Figure 2c). C_4_ mid‐grass production was 3–4 times greater in top‐kill than woodland in fall 2010 and 2011, declined in both treatments to near zero in 2012–2013, and was 4–25 times greater in top‐kill than woodland in 2014–2015 (Figure 2d).

**Figure 3:**
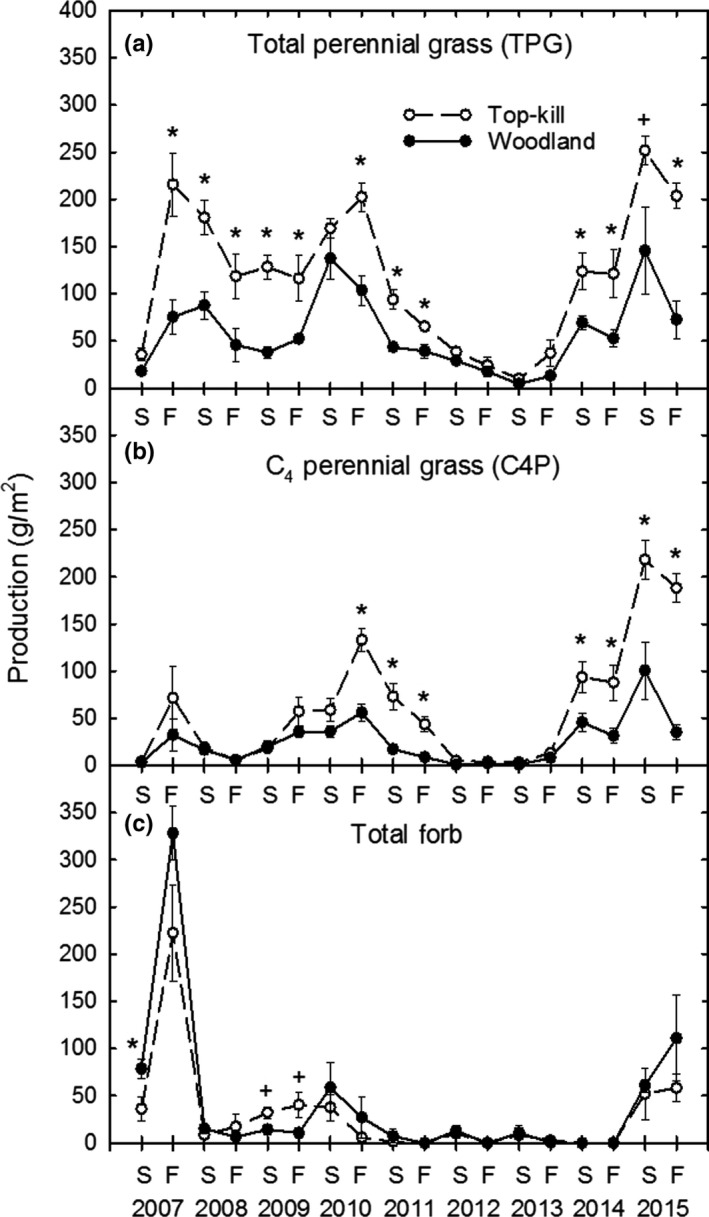
Total perennial grass  (a), C_4_ perennial grass  (b), and total forb (c) production in interspaces in top‐kill and woodland treatments by summer (S) and fall (F) each year. Asterisk and plus signs same as Figure 2

Total perennial grass production was nearly three times greater in top‐kill than woodland by fall of the first year post‐treatment, remained greater until the drought of 2012, and was again greater in top‐kill than woodland in 2014 and 2015 (Figure 3a). C4P production was greater in top‐kill than woodland in 2010–2011 and in 2014–2015, after the drought (Figure 3b). Total forb production remained low in both treatments, except for a pulse (mostly *A. dracunculoides*) in both treatments in 2007 (Figure 3c).

**Figure 4:**
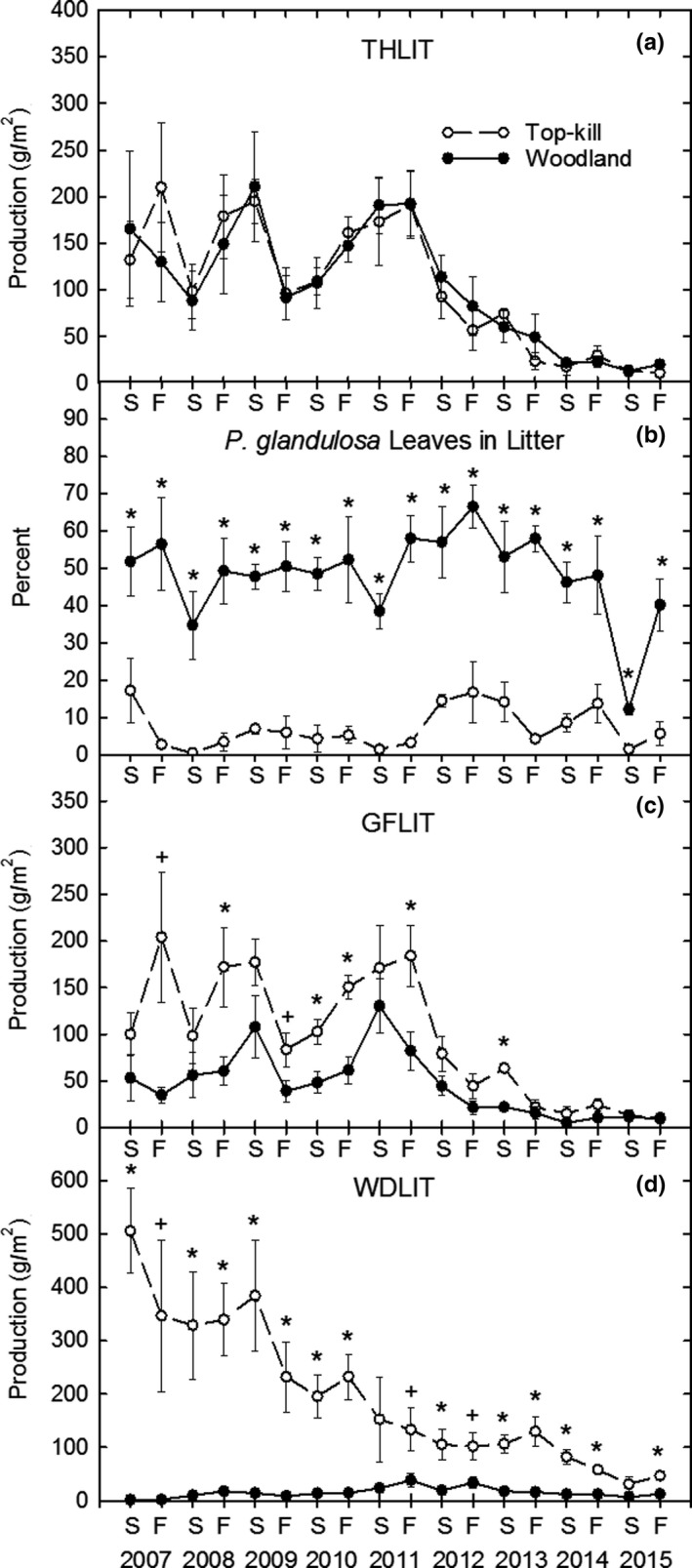
THLIT (a), GFLIT (c), and WDLIT (d) production, and percent *P. glandulosa *leaves in litter (b) in interspaces in top‐kill and woodland treatments by summer (S) and fall (F) each year. Asterisk and plus signs same as Figure 2

Total herbaceous litter production was not different between treatments during the study, declined beginning in 2012 and did not increase in either treatment for the remainder of the study (Figure 4a). The percent of THLIT that was *P. glandulosa* leaf was greater in woodland on all sample dates (Figure 4b). GFLIT was greater in top‐kill than woodland mostly in the first 5 years (Figure 4c). WDLIT initially was greater in top‐kill than woodland, but declined to near zero in both treatments by 2015 (Figure 4d).

### 3.3 Treatment and microsite effects on herbaceous cover

**Figure 5:**
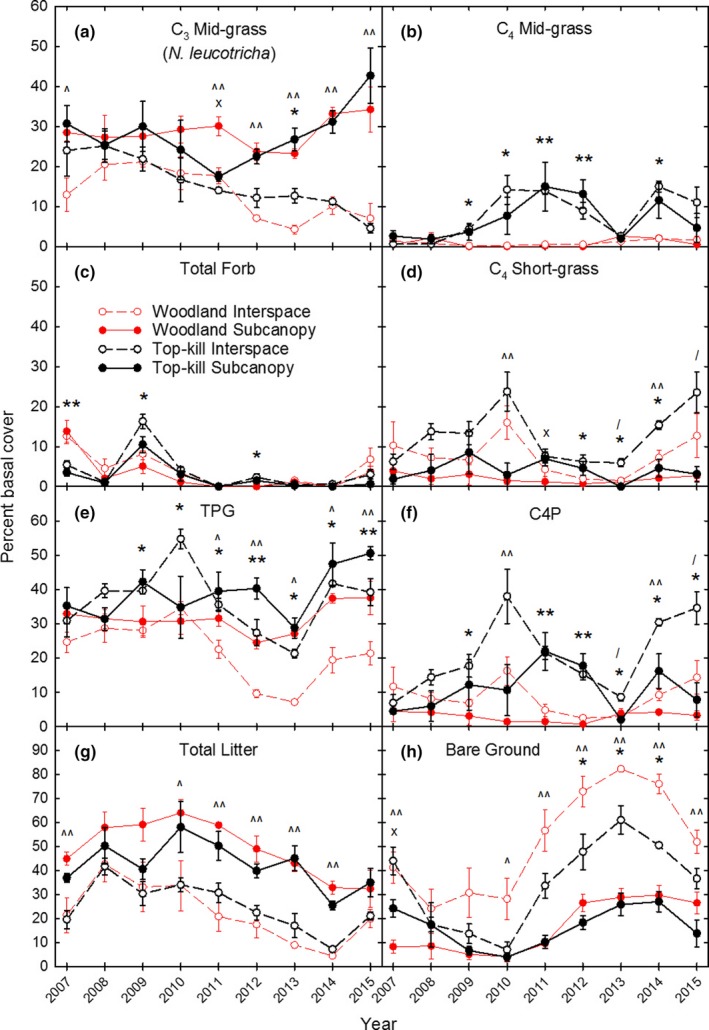
Percent basal cover of C_3_ mid‐grass (a), C_4_ mid‐grass (b), total forb (c), C_4_ short‐grass (d), TPG (e), C4P (f), total litter (g) and bare ground (h) in top‐kill versus woodland treatment and interspace versus subcanopy microsites each year. Symbols represent differences at *p* ≤ .05 for the following comparisons: ** = Different between woodland versus top‐kill in both interspace and subcanopy (red vs. black); * = Different between woodland versus top‐kill in interspace only (red dashed vs. black dashed); × = Different between woodland versus top‐kill in subcanopy only (red solid vs. black solid); ^^ = Different between interspace versus subcanopy in both woodland and top‐kill (solid vs. dashed); ^ = Different between interspace versus subcanopy in woodland only (solid red vs. dashed red); / = Different between interspace versus subcanopy in top‐kill only (solid black vs. dashed black)

C_3_ mid‐grass (*N. leucotricha*) cover in interspaces declined in both treatments during the study and was greater in the top‐kill than the woodland treatment only in 2013 (Figure 5a). C_3_ mid‐grass cover in subcanopy was similar in both treatments from 2007 to 2010, declined sharply in top‐kill, but not woodland, in 2011, and steadily increased in both treatments from 2013 to 2015. C_4_ mid‐grass cover in interspaces was greater in top‐kill than woodland in 5 of the 9 years (2009–2012, 2014; Figure 5b). In the subcanopy microsite, C_4_ mid‐grass cover did not become greater in top‐kill compared to woodland until 2011 and remained so only through 2012.

Forb basal cover was greater in woodland than top‐kill in both microsites in 2007 and was greater in top‐kill than woodland in interspaces in 2009 and 2012 (Figure 5c). C_4_ short‐grass cover in interspaces was greater in top‐kill than woodland from 2012 to 2014, but in subcanopy was greater in top‐kill than woodland only in 2011 (Figure 5d).

Total perennial grass cover in interspaces was greater in top‐kill than woodland in 7 of 9 years (2009–2015), but in subcanopy was greater in top‐kill than woodland in only 2012 and 2015 (Figure 5e). C4P cover in interspaces was greater in top‐kill than woodland in 6 of 9 years (2009, 2011–2015), but in subcanopy was greater in top‐kill than woodland in only 2011 and 2012 (Figure 5f).

Total litter cover in interspaces declined in both treatments from 2008 to 2014, and in subcanopy, declined in both treatments from 2010 to 2014 with no differences between treatments in any year (Figure 5g). Percent bare ground in interspaces was greater in the woodland than the top‐kill treatment in 3 years (2012–2014). Bare ground in subcanopy was greater in top‐kill than woodland in 2007 (Figure 5h).

Regarding microsite effects, C_3_ mid‐grass cover remained stable in woodland subcanopy throughout all years but declined in woodland interspaces after the drought (2012–2015; Figure 5a). C_3_ mid‐grass cover in the top‐kill treatment decreased in both microsites during 2007–2011, but increased in subcanopy while continuing to decrease in interspaces from 2012 to 2015.

C_4_ mid‐grass cover in woodland remained <4% in both microsites for the entire study period, but increased to a peak of 16% in the top‐kill treatment and followed the same year‐to‐year pattern in both microsites, with the exception that peak cover occurred 1 year earlier in interspaces (2010) than in subcanopy (2011; Figure 5b). Following the drought, C_4_ mid‐grass cover increased in both top‐kill microsites in 2014 due to the >200% above normal July precipitation in 2013 and 2014. Forb cover was similar between microsites in both top‐kill and woodland treatments in all sampling years (Figure 5c). C_4_ short‐grass cover was greater in interspaces than subcanopy in both treatments in 2010 and 2014 and in only the top‐kill treatment in 2013 and 2015 (Figure 5d).

Total perennial grass cover was greater in subcanopy than interspace microsites in both treatments in 2012 and 2015 and in woodland only in 2011, 2013, and 2014 (Figure 5e). C4P cover was greater in interspace than subcanopy in both treatments in 2010 and 2014 and only in the top‐kill treatment in 2013 and 2015 (Figure 5f). Total litter cover was greater in subcanopy than interspaces in both treatments in 2007 and 2011–2014 and in woodland only in 2010 (Figure 5g). Percent bare ground was much greater in interspace than subcanopy microsites in both treatments in 2007 and 2011–2015 and in woodland only in 2010 (Figure 5h).

**Figure 6:**
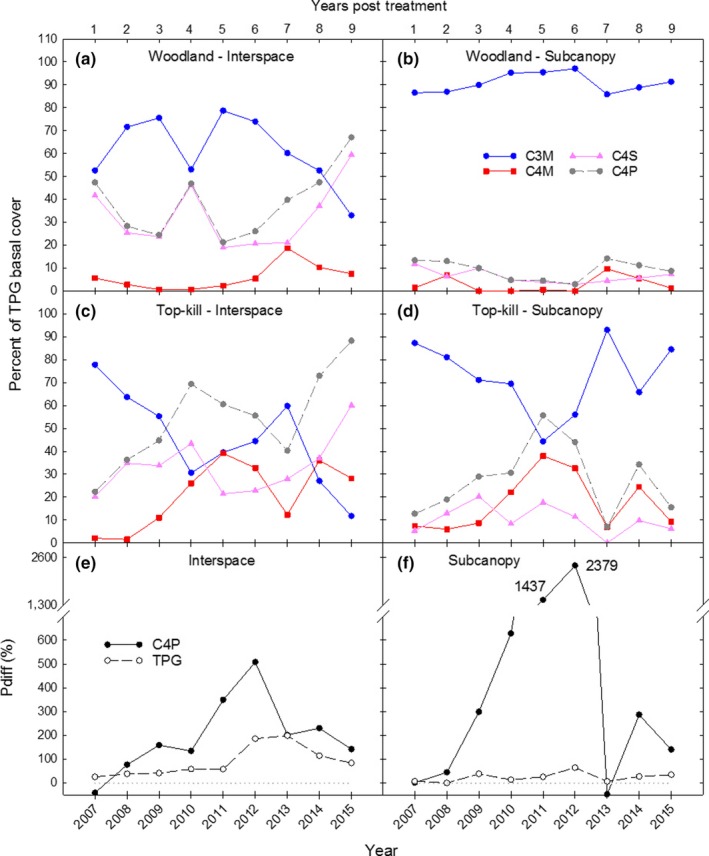
Percent of total perennial grass (TPG) basal cover that is C_3_ mid‐grass (C3M), C_4_ mid‐grass (C4M), C_4_ short‐grass (C4S), and C_4_ perennial grass (C4P) within interspace and subcanopy in woodland and top‐kill (a–d), and Pdiff for C4P and TPG cover in interspace and subcanopy each year (e, f) (Pdiff was derived from values in Figure 5e,f)

In comparing grass functional group cover as a percentage of TPG, in woodland interspaces, C_3_ mid‐grasses dominated in most years, C_4_ short‐grasses accounted for most of the cover of C4P, and C_4_ mid‐grasses were low in all years with a slight rise in 2013 (Figure 6a). In woodland subcanopy, C_3_ mid‐grasses dominated in all years with C4P never exceeding 15% cover (Figure 6b). In top‐kill interspaces, C4P increased relative to C_3_ mid‐grasses throughout the study, the only exception being 2013 (Figure 6c). C_4_ short‐grasses accounted for most of the C4P cover in most years. By 2015, there was a much greater separation between C_3_ mid‐grasses and C4P in top‐kill interspaces than was found in woodland interspaces. Within top‐kill subcanopy, C_3_ mid‐grass dominance gradually declined from 2008 to 2011, while C4P (mostly C_4_ mid‐grasses) increased (Figure 6d). The drought in 2011–2013 reversed this trend beginning in 2012.

The percent difference in basal cover means between top‐kill and woodland was much greater for C4P than for TPG in most years and the difference for C4P was much greater in subcanopy than interspace microsites until 2013 when the C4P value in subcanopy sharply declined to below zero (Figure 6e,f).

## 4 DISCUSSION

### 4.1 Woody plant facilitation of C_3_ grasses

The *Prosopis*/*Nasella* example of woody species facilitation of C_3_ grass expansion in C_4_ grassland is a similar process to that described in south Texas, where a low‐to‐moderate canopy cover of the woody legume *Acacia smallii* increased *N. leucotricha* growth compared to no woody cover (Scifres, Mutz, Whitson, & Drawe, 1982). Fuhlendorf, Smeins, and Taylor (1997) found that *N. leucotricha* was the most common grass species beneath evergreen *Juniperus ashii* trees in a C_4_ grassland in central Texas. However, this occurred only beneath trees that had lower portions of canopies removed from goat browsing, thus allowing enough light penetration for *N. leucotricha*, but not C_4_ mid‐grass, growth. In the Sonoran Desert of central Arizona, Schade, Sponseller, Collins, and Stiles (2003) found more C_3_ annual grasses beneath than outside *Prosopis velutina* canopies.

Examples of woody species facilitating the advance of C_3_ grasses in C_4_ grasslands occur worldwide. Rossi and Villagro (2003) described a vegetation complex in central Argentina similar to our study in which 4–6 m tall *Prosopis flexuosa* facilitated growth of the unpalatable C_3_ grass *Stipa ichu* beneath the *P. flexuosa* canopy, while C_4_ grasses were more frequent in interspaces. Rauber et al. (2014) found in semiarid *Prosopis caldenia* forests of central Argentina that native unpalatable C_3_ grasses, *S. ichu* and *Nassella tenuissima*, expanded in C_3_/C_4_ mixed grassland that had previously been mostly C_4_ grasses. They did not mention that *P. caldenia* facilitated this process; however, their statistical analysis showed a strong association between *P. caldenia* and these two grass species. C_3_ afforestation projects in C_4_ grasslands of Argentina have documented increases in C_3_ grasses beneath planted poplar trees (Nordenstahl, Gundel, Clavijo, & Jobbagy, 2011).

In South Africa, Stuart‐Hill and Tainton (1989) described a grass community beneath *Acacia karoo* that had become dominated by C_3_ *Cymbopogon plurinodis* to the exclusion of C_4_ *Themeda triandra*. In Australia, Prober et al. (2005) used carbon supplements to shift woodland understories that had become dominated with exotic C_3_ annual grasses or the native C_3_ perennial grass *Poa sieberiana* toward the native presettlement C_4_ grass, *Themeda australis*.

### 4.2 Grass and litter responses to *Prosopis* top‐kill

Our study demonstrated that top‐killing suppression of the *P. glandulosa* overstory in a C_3_/C_4_ mixed grassland that had become dominated by a C_3_ mid‐grass (*N. leucotricha*) in lieu of C_4_ mid‐grasses, initially stimulated C_3_ mid‐grass production and later increased C_4_ mid‐grass production. C_4_ short‐grass production was unaffected by *P. glandulosa* top‐kill. Laxson et al. (1997) predicted that such a shift between C_3_ mid‐grass and C_4_ mid‐grass production would occur following *P. glandulosa* treatment, but their 2‐year study was not long enough to verify this.

We also demonstrated that the window of opportunity for C_4_ mid‐grasses to displace C_3_ mid‐grasses was quite small. C_4_ mid‐grasses first had to withstand the initial surge of C_3_ mid‐grass growth, then had only a few years to increase production and basal cover before *P. glandulosa* regrowth began to limit C_4_ mid‐grass growth. The window for C_4_ mid‐grass recovery narrowed even further with the severe drought in years 5 and 6, just as C_4_ mid‐grass production and cover had increased to levels similar to C_3_ mid‐grasses.

A historically rare occurrence of high July rainfall in 2013 and 2014 stimulated C_4_ mid‐grass production in the top‐kill treatment to its greatest level (130 g/m^2^) during the study. However, this remained well below 400 g/m^2^ production potential for this grass group (Ansley et al., 2013). Suboptimal C_4_ mid‐grass production in this treatment was due to several factors. First, *P. glandulosa* regrowth was likely directly competing with C_4_ mid‐grasses for water via lateral roots that extended into interspaces. Ansley et al. (2018) found that during the 2011–2012 drought, regrowth *P. glandulosa* extracted soil moisture from interspaces at a greater rate than did woodland *P. glandulosa*. Second, the region was recovering from drought and percent bare ground remained high. The greater increase in bare ground in interspace than subcanopy microsites in the top‐kill treatment from 2010 to 2012 (Figure 5g,h) illustrates the competitive effects of mesquite lateral roots in interspaces. Finally, litter production and cover that would have buffered soil temperature extremes and limited evaporation loss of soil moisture were very low. This may have negatively affected emergence of new C_4_ mid‐grass propagules.

During years 1–5, the increase in GFLIT production in top‐kill interspaces compared to woodland (Figure 4c) was likely due to increased grass production. However, the drought initiated a decline in THLIT and GFLIT production as well as total litter cover in interspaces in both treatments. While THLIT and GFLIT production never recovered, total litter cover increased slightly from <10% to 20% in both treatments in 2015. This increase may have been due to a small amount of grass leaves or *P. glandulosa* leaves that could have increased surface cover without adding much mass to THLIT production.

### 4.3 Stability of the *Prosopis*/*Nassella* association

**Figure 7:**
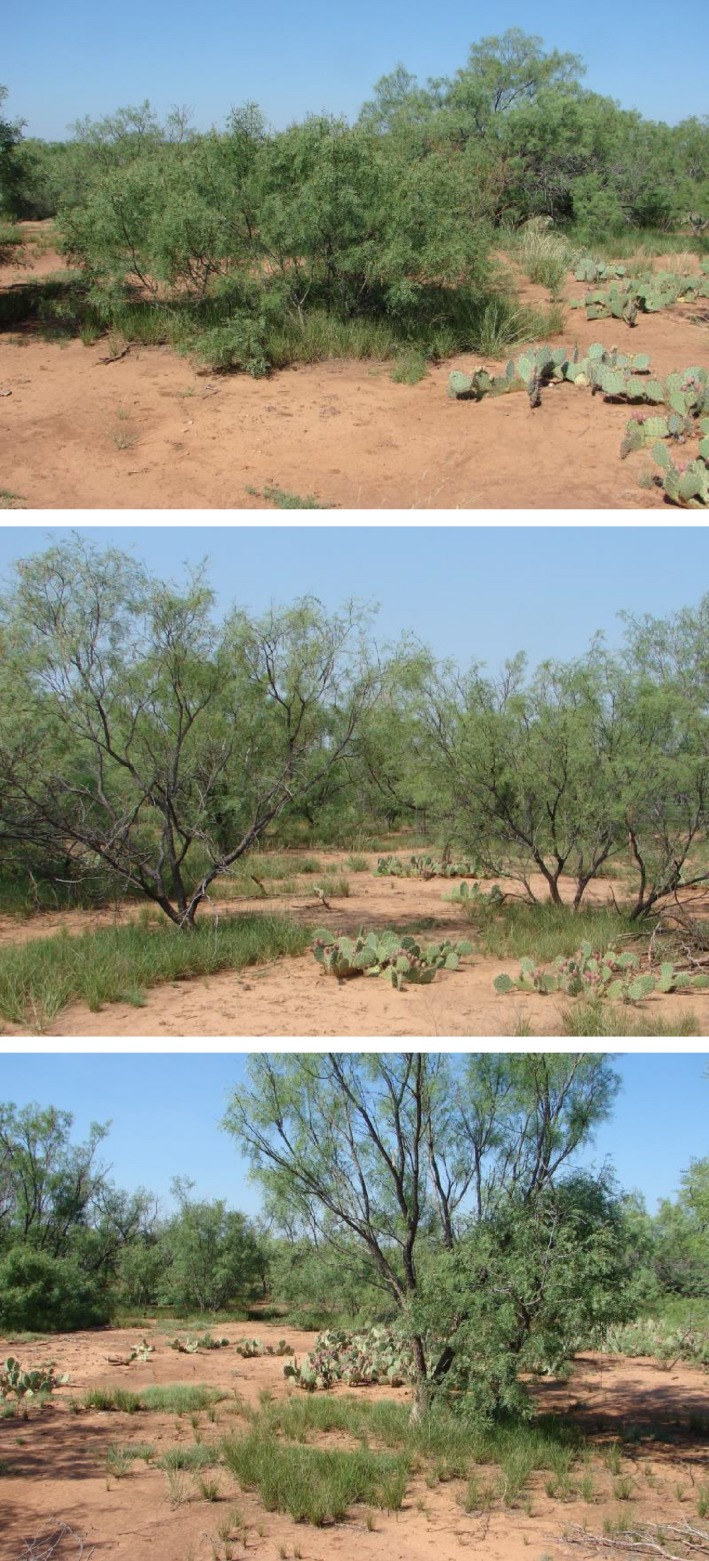
*Prosopis glandulosa* of varying heights with a *Nassella leucotricha* understory in July 2014, following the severe drought of 2011–2012. Interspaces are mostly bare ground and pricklypear cactus (*Opuntia* spp)

Basal cover responses of *N. leucotricha* illustrate the close association of this species with *P. glandulosa*. In untreated woodland, the observation that *N. leucotricha* cover remained stable in the subcanopy microsite during the severe drought indicates that *P. glandulosa* canopies serve as *refugia* for *N. leucotricha*, but not C_4_ grasses, during drought (Figures 6b and 7). Within the top‐kill subcanopy microsite, *N. leucotricha* basal cover declined relative to C_4_ mid‐grass cover during the first 5 years after top‐kill. However, the advantage shifted back to *N. leucotricha* in years 6 through 9 (Figure 6d) as canopy area of regrowth *P. glandulosa* increased from 8.5 to 14.7 m^2^/tree (Ansley et al., 2018). At 578 trees/ha, by year 7 (canopy area 12.6 m^2^/tree), more than half of the land area had again become subcanopy microsite that was more suitable for *N. leucotricha* than C_4_ mid‐grass growth. These results suggest that after *N. leucotricha* establishes beneath *P. glandulosa* canopies, a single top‐killing disturbance would have little effect on shifting subcanopy composition toward C_4_ mid‐grasses.

Regarding grazing impacts, persistence of the *Prosopis*/*Nassella* association is strengthened because, unlike C_4_ mid‐grasses, *N. leucotricha* is dormant and unpalatable during summer months and therefore receives little grazing pressure. It is palatable and grazed in early spring, but recovers well because there is no drought or competition from *P. glandulosa*. In contrast, C_4_ mid‐grasses grow in late spring and summer when they are simultaneously exposed to grazing pressure, competition from *P. glandulosa* and drought.

C_4_ short‐grass production did not increase following *P. glandulosa* top‐kill, although basal cover increased slightly in interspaces. Similarly, Ansley et al. (2013) found no relation between changes in *P. glandulosa* canopy cover and C_4_ short‐grass production. Thus, it appears that C_4_ short‐grasses remain in this ecosystem at some small level, mostly in interspaces than subcanopy, but do not supplant C_3_ mid‐grasses or C_4_ mid‐grasses. When *P. glandulosa* is low density or not present, average annual precipitation is apparently sufficient to allow C_4_ mid‐grasses rather than C_4_ short‐grasses to dominate the herbaceous layer. Under advanced *P. glandulosa* invasion, C_3_ mid‐grasses (*N. leucotricha*) dominate instead of C_4_ short‐grasses. C_4_ short‐grasses are very drought and grazing tolerant, and this partially explains why they persist under a wide range of mid‐grass and *P. glandulosa* cover.

Heitschmidt, Schultz, and Scifres (1986), Belsky et al. (1989), Ansley and Castellano (2006), and others have noted the positive aspects of the presence of some trees in C_4_ grassland as a savanna. They argue that it promotes species diversity and guards against drought. However, as we better understand the consequences of invasion by deciduous, resprouting woody legumes, such as *P. glandulosa*, all of these attributes result in the advancement of C_3_ grasses or possibly other C_3_ herbaceous species, to the detriment of C_4_ grasses. The perception of *P. glandulosa* as a desired *refugia* during drought, as Figure 7 might suggest to a casual observer, represents a misinterpretation of the factors preceding the dominance of the *Prosopis*/*Nassella* association over C_4_ grasses and an inability of top‐killing disturbances to affect transition back to a C_4_ grassland state prior to drought occurrence.

Bond and Midgley (2000) predicted that normal windows for C_4_ mid‐grass recovery after woody plant suppression may shorten in the future if woody regrowth rates increase with increasing atmospheric CO_2_. The “fire‐trap” concept (Bond & Keeley, 2005; Freeman et al., 2017) emphasized that strong woody resprouters after top‐killing fires limit grass recovery unless fire disturbances are of sufficient frequency. These studies did not consider the C_3_ grass factor shown in this study that narrows the C_4_ grass recovery window to an even greater degree. The *Prosopis*/*Nassella* association may be too stable for even multiple woody suppression events to affect transition to C_4_ grass dominance, especially under projected changes in climate and CO_2_ concentration.

**Figure 8:**
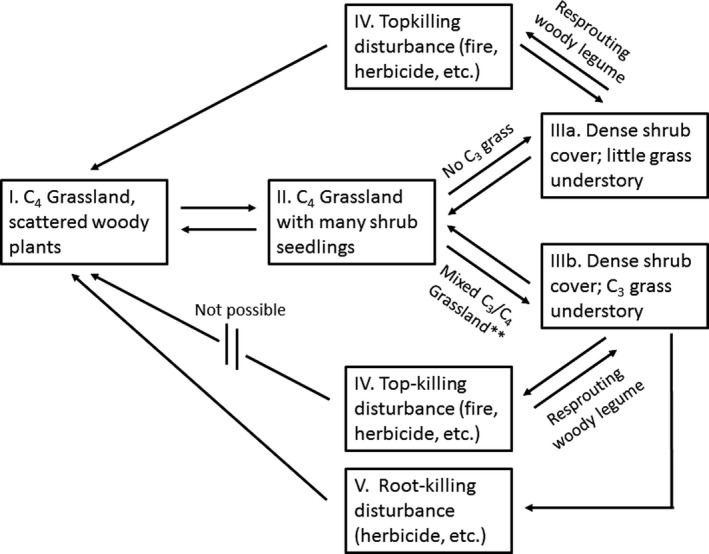
Modification of state‐and‐transition model presented by Westoby et al. (1989) and Briske et al. (2003) (I, II, IIIa, and IV), and a new component that is specific to the SGP showing how the *Prosopis*/*Nassella* association (IIIb) prevents transition to C_4_ grassland (I) if *Prosopis glandulosa* is only top‐killed (IV). Transition may be possible if *P. glandulosa* is root‐killed via anthropogenic inputs (V). **Mixed C_3_/C_4_ grassland that either had originally a small component of C_3_ grass *Nassella leucotricha* or was invaded by exotic C_3_ perennial grasses

Persistence of the *Prosopis*/*Nassella* association, or similar associations, may radically affect disturbance‐based state‐and‐transition models in C_3_/C_4_ mixed grassland ecosystems that are invaded by deciduous resprouting woody legumes. Once woody density exceeds a threshold, treatments that cause whole plant mortality of the woody component may be the only solution to affect a true transition from C_3_ woody/C_3_ grass back to C_4_ grassland, as we demonstrate with a modification to the state‐and‐transition model presented by Westoby et al. (1989) (Figure 8). In the future, more detailed rangeland state‐and‐transition models (e.g., Bashari, Smith, & Bosch, 2009) may include woody canopy effects on C_3_/C_4_ grass mixes.

## CONFLICT OF INTEREST

None declared.

## AUTHOR CONTRIBUTIONS

R.J.A. designed and executed the study. R.J.A. and C.C. wrote the manuscript. T.Z. assisted with data collection and manuscript writing.

## Data Availability

Production and cover response data: Dryad https://doi.org/10.5061/dryad.8w9ghx3gp.
